# Mixed Green Banana (*Musa* spp.) Pulp and Peel Flour Reduced Body Weight Gain and Adiposity and Improved Lipid Profile and Intestinal Morphology in *Wistar* Rats

**DOI:** 10.3390/nu17152493

**Published:** 2025-07-30

**Authors:** Leonara Martins Viana, Bárbara Pereira da Silva, Fabiana Silva Rocha Rodrigues, Laise Trindade Paes, Marcella Duarte Villas Mishima, Renata Celi Lopes Toledo, Elad Tako, Hércia Stampini Duarte Martino, Frederico Barros

**Affiliations:** 1 Department of Food Technology, Federal University of Viçosa, Viçosa 36570-900, MG, Brazil; leonara.viana@ufv.br (L.M.V.); fabiana.s.rodrigues@ufv.br (F.S.R.R.); laise.paes@ufv.br (L.T.P.); fredbarros@ufv.br (F.B.); 2 Department of Nutrition and Health, Federal University of Viçosa, Viçosa 36570-900, MG, Brazil; barbarapereira2805@gmail.com (B.P.d.S.); marcella.mishima@ufv.br (M.D.V.M.); renatacelly@yahoo.com.br (R.C.L.T.); hercia@ufv.br (H.S.D.M.); 3 Department of Food Science, Cornell University, Stocking Hall, Ithaca, NY 14850, USA

**Keywords:** bioactive compounds, intestinal functionality, dietary fiber, resistant starch, unripe banana flour, gene expression

## Abstract

**Background and Objectives:** In recent years, there has been growing interest in the production of ingredients rich in dietary fiber and antioxidants, such as green banana flours. This study evaluated the effect of consumption of mixed green banana pulp (PF) and peel (PeF) flours on the body weight gain, adiposity, lipid profile, and intestinal morphology of *Wistar* rats. **Methods:** Male young rats were divided into four groups (n = 8) that received a standard diet (SD), or one of the following three test diets: M1 (SD + 90% PF/10% PeF), M2 (SD + 80% PF/20% PeF), or P (SD + 100% PF) for 28 days. **Results:** Rats from M1, M2, and P groups showed reduced body weight gain and adiposity and had lower contents of total cholesterol, LDL-c, VLDL-c, and triglycerides. Animals from M1 and M2 groups had an increase in cecum weight, fecal moisture, acetic acid concentration, and crypt depth and reduced fecal pH. Moreover, consumption of the M1, M2, and P diets increased the expression of proteins involved in intestinal functionality. Significant negative correlations were observed between consumption of resistant starch and soluble dietary fiber, from the flours, and weight gain (r = −0.538 and r = −0.538, respectively), body adiposity (r = −0.780 and r = −0.767, respectively), total cholesterol (r = −0.789 and r = −0.800, respectively), and triglycerides (r = −0.790 and r = −0.786, respectively). **Conclusions:** Mixed green banana pulp and peel flour proved to be a viable alternative as a food ingredient that can promote weight loss, improve lipid profile and intestinal morphology, and minimize post-harvest losses.

## 1. Introduction

In recent years, there has been growing interest in the production and consumption of convenient food products that provide beneficial nutritional and functional properties [1]. Therefore, the consumption of foods rich in resistant starch has attracted attention due to their recognized health benefits. Resistant starch naturally occurs in foods and can be found in grains, raw potatoes, green bananas, or even in retrograde starchy products [2,3].

Banana is one of the most consumed fruits in the world, being produced mostly in tropical regions [4]. In addition to its high nutritional value, banana plays an important role in food security and as an economic base in several countries, which implies high consumption by the population [4,5]. However, as it belongs to one of the classes of perishable fruits, and since it presents early deterioration during the handling, storage, and transport stages, interest in flours derived from alternative ingredients such as green banana pulp and peel has grown significantly.

The high concentration of resistant starch in green banana pulp flour has been linked to benefits related to glucose tolerance and a decrease in serum levels of cholesterol and triglycerides, in addition to contributing to the prevention of cardiovascular diseases [6,7]. Several studies have also attributed an increased satiety and micronutrient absorption to the dietary consumption of green banana pulp, as well as its role as a substrate for the production of short-chain fatty acids (SCFA) [6,8,9].

Green banana peel flour has high concentrations of minerals, dietary fiber, and phenolic compounds [10,11], and it may have a positive impact on intestinal health, since these compounds act to improve intestinal functionality and morphology. In addition, one of the ways to reduce banana waste is to use the peels to produce flour, which has been considered a possibility of innovation for the food industry, as it has the potential to contribute to waste reduction and may also be useful in the production of sustainable foods with high additional nutritional value and low cost.

Thus, it is believed that mixed green banana pulp and peel flours are able to improve the nutritional value, contribute to diversified formulations of food products, and reduce banana waste. Furthermore, to date, there are no studies that have evaluated the effects of consumption of mixed green banana pulp and peel flour on adiposity, lipid profile, blood glucose levels, and intestinal parameters, in vivo. Therefore, the objective of this study was to evaluate the effects of the dietary consumption of mixed green banana pulp (PF) and peel (PeF) flours on body weight gain, adiposity, lipid profile, and intestinal morphology of young *Wistar* rats.

## 2. Materials and Methods

### 2.1. Raw Materials and Preparation of Banana Flours

Green bananas (*Musa* spp.) of the ‘Prata’ variety were sanitized with chlorinated solution (100 mg/L for 20 min) and manually peeled. Then, unripe banana peel was promptly subjected to immersion in a 0.5% (*w*/*v*) citric acid solution, and the pulp was immersed in potable water [11]. This process was maintained for about 20 min to minimize enzymatic browning. Subsequently, green banana pulp and peel were sliced (2 mm) and dehydrated in a tray dryer (Polidryer Model PD 150, Viçosa, Brazil) with forced air circulation of 1.5 m/s at a constant temperature of 55 °C for 9 h. The dried material was ground (Brabender, model WI, Duisburg, Germany) and sieved in 1.0 mm sieves. The mixed flours were obtained by manually mixing green banana pulp and peel flours in the ratios (pulp–peel) of 90:10 (M1) and 80:20 (M2) for 1 min in a plastic container [11] and were stored at freezing temperature (−18 °C).

### 2.2. Proximate Composition

All analyses were carried out following the official methods established by the Association of Official Analytical Chemists [12]. Moisture content was determined using a hot air circulation oven at 100–105 °C for 4 h. Protein content (%N × 6.25) was measured using the Kjeldahl method. Total lipid content was assessed via Soxhlet extraction with petroleum ether (40–60 °C). Ash content was determined by incineration in a muffle furnace at 550 °C. Total carbohydrate content was calculated the difference of 100 − (moisture + ash + protein + lipids). Total dietary fiber, including soluble and insoluble fractions, was quantified using a gravimetric–enzymatic method with a commercial assay kit (Total Dietary Fiber Assay Kit, Sigma, St. Louis, MO, USA).

### 2.3. Total Phenolic Content

The content of total phenolic compounds was determined using the method described by Singleton and Rossi (1965) [13], with minor modifications. For extraction, 500 mg of flour was mixed with 25 mL of methanol and stirred continuously for 2 h. The mixture was then centrifuged at 4000× *g* for 15 min. An aliquot of 0.1 mL of the supernatant was combined with 0.4 mL of Folin–Ciocalteu reagent (previously diluted with distilled water), 0.9 mL of ethanolamine solution, and 1.1 mL of distilled water. The total phenolic content was expressed as milligrams of gallic acid equivalents per gram of flour (mg GAE/g).

### 2.4. Total Starch and Resistant Starch Contents

Total starch and resistant starch contents were determined according to the American Association of Cereal Chemists (AACC) methods 76–13 and 32–40, respectively [14]. The analyses involved simulated enzymatic digestion using pancreatic α-amylase and amyloglucosidase, following the procedures outlined in a commercial assay kit (Megazyme International, Wicklow, Ireland) and adhering to the manufacturer’s instructions.

### 2.5. In Vivo Study

#### 2.5.1. Experimental Model

Thirty-two male rats (*Rattus norvegicus*, *Wistar*, albinus variation), recently weaned (21 days old) from the Central Animal Facility of the Center for Biological Sciences and Health at the Federal University of Viçosa (Minas Gerais, Brazil), were systematically subdivided into four groups with eight animals each, randomized by body weight. The number of animals per group (n) was calculated based on the sample calculation equation, assuming a significance level of α = 0.05 (type I error) and a corresponding Z-score of 2.04. The rats were distributed into individual stainless-steel cages and kept at 12 h/12 h on light and dark cycles at an average temperature of 22 °C. The animals received access to deionized water and their respective experimental diets ad libitum.

The experimental diets (Table 1) were formulated based on the AIN-93G standard diet [15], with partial or total substitution of corn starch and dextrinized starch by green banana pulp flour and blends of green banana pulp and peel flours, in ratios of 90:10 and 80:20 (pulp–peel). These proportions were selected based on previous research conducted by our group, which demonstrated that incorporating up to 20% peel flour into pulp flour does not adversely affect the technological properties of the starch [11].

Each group received one of the following experimental diets: standard diet (SD, AIN-93G diet); mixture 1 (M1, standard diet + 90% of the PF and 10% of the PeF); mixture 2 (M2, standard diet + 80% of the PF and 20% of the PeF), or pulp flour (P, standard diet + 100% of the PF). The concentration of green banana pulp flour and the mixtures of green banana pulp and peel flours (M1 and M2) was calculated based on the total dietary fiber in the control diet, so that the test diets (P, M1 and M2 groups) provided 100% of the total dietary fiber content, of that provided by the AIN-93G diet. Then, the amount of these flours in the experimental diets was calculated according to their respective compositions (Table 2). All other ingredients were added in sufficient quantities to provide the planned amount of lipids, proteins, carbohydrates, total dietary fiber, and calories.

On the 28th day, following a 12 h fasting period, the animals were anesthetized using isoflurane (Isoforine^®^, Cristália, Itapira, Brazil), and blood was collected via cardiac puncture into dry tubes. The blood samples were centrifuged at 2865× *g* for 10 min (Fanem-204, São Paulo, Brazil), and the resulting serum was stored at −80 °C. Feces, duodenum, and adipose tissue were collected, weighed, and similarly stored at −80 °C. The cecum was excised and preserved in 10% formaldehyde at room temperature for subsequent histological analysis. Throughout the experimental period, body weight gain and food intake were monitored weekly. Consumption of dietary fiber (both soluble and insoluble fractions), resistant starch, and total phenolics was calculated by multiplying the average daily food intake by the amount of these compounds in the diet. The study protocol was approved by the Ethics Committee on Animal Research of the Federal University of Viçosa (Protocol No. 21/2021; approved on 30 April 2021).

#### 2.5.2. Food Efficiency Ratio, Cecum Index, and Total Body Adiposity

The feed efficiency ratio (FER) was calculated by the ratio between body weight gain and total diet consumption [16]. The cecum index was calculated as a ratio between cecum weight (g) and total body weight (g), multiplied by 100 [17]. Adiposity was calculated as a percentage using the following formula: (visceral + retroperitoneal + epididymal + abdominal adipose tissues)/total body weight × 100 [18].

#### 2.5.3. Biochemical Analyzes

For the determination of biochemical parameters, 0.5 mL of serum was used. Serum glucose concentrations, total cholesterol, high-density lipoprotein cholesterol (HDL-c), low-density lipoprotein cholesterol (LDL-c), very low-density lipoprotein (VLDL-c), triglycerides (TGL), and uric acid levels and aspartate aminotransferase (AST) and alanine aminotransferase (ALT) activities were measured by colorimetric methods using commercially available kits (Bioclin^®^, Belo Horizonte, Brazil). Analyses were conducted on a BS-200 Chemistry Analyzer (Bioclin^®^).

#### 2.5.4. Cecum Histomorphometry Analysis

Semi-serial histological sections of the cecum, 3 μm thick, were obtained using an automated rotary microtome (Reichert-Jung^®^, Genossen, Germany) and stained with hematoxylin and eosin. The slides were examined under a light microscope (Olympus CX40, Olympus, Tokyo, Japan), and digital images were captured using a photomicroscope (Olympus AX70 TRF, Tokyo, Japan). For morphometric analysis—including measurements of crypt depth, crypt thickness, goblet cell count, and the thickness of the circular and longitudinal muscle layers—twenty random fields per animal were selected. Only crypts with clearly defined and intact epithelial linings were included, as described by Cavaliere (2013) [19]. Image analysis was performed using ImagePro-Plus^®^ software, version 4.5 (Media Cybernetics, Rockville, MD, USA).

#### 2.5.5. Fecal pH

For the analysis of fecal pH, about 0.5 g of the cecum content was homogenized in 5 mL of distilled water (1:10, g:mL), with the aid of a vortex (Kasvi^®^, Jiangyan city, China). The pH was measured with a calibrated digital pH meter (Bel Engineering^®^, Monza, Italy) until the pH stabilized [20].

#### 2.5.6. Fecal Moisture Content

The moisture content in the feces was determined by the gravimetric method using an oven with forced air circulation (Nova Ética^®^, 400/6ND, Sao Paulo, Brazil) at 105 °C for 24 h, according to the methodology proposed by the AOAC (2016) [12].

#### 2.5.7. Short-Chain Fatty Acids (SCFA)

Approximately 100 mg of cecal content was homogenized in 300 μL of Milli-Q water using a vortex mixer and centrifuged at 12,000× *g* for 10 min. The supernatant was collected, and subsequent procedures were carried out according to the method described by Siegfried, Ruckemmann, and Stumpf (1984) [21]. Short-chain fatty acid (SCFA) concentrations were quantified using High-Performance Liquid Chromatography (HPLC) (Prominence Shimadzu LC-20A, Shimadzu Corporation, Kyoto, Japan), equipped with an RID-20A refractive index detector and an Aminex HPX-87H column (300 × 7.8 mm, 8%; Bio-Rad, Hercules, CA, USA). The mobile phase consisted of 5 mM of sulfuric acid (H_2_SO_4_) in ultrapure water. Each sample (20 μL) was injected under isocratic conditions, with a flow rate of 0.7 mL/min at a column temperature of 45 °C. Acetic, propionic, and butyric acids (Sigma-Aldrich, São Paulo, Brazil) were used as external standards for calibration.

#### 2.5.8. Extraction of mRNA from Intestinal Tissue and cDNA Synthesis

Intestinal tissue (duodenum) was macerated in liquid nitrogen under RNAse free conditions, and the samples were aliquoted for total RNA extraction. Total RNA was extracted with the TRIzol Reagent (Invitrogen, Carlsbad, CA, USA). The extracted mRNA was used to synthesize the cDNA with the M-MLV reverse transcription kit (Invitrogen Corp., Grand Island, NY, USA) [22].

#### 2.5.9. Determination of Gene Expression of Proteins Involved in Intestinal Health by Quantitative Reverse Transcriptase Polymerase Chain Reaction (RT-qPCR)

mRNA expression levels of genes in the intestinal tissue that are involved in intestinal health were analyzed by RT-qPCR. The SYBR Green PCR master mix from Applied Biosystems (Foster City, CA, USA) was employed, and the analyses were performed on the StepOne™ Real-Time PCR System (Thermo Fisher Scientific, Waltham, MA, USA) by means of the measurement system involving SYBR-Green Fluorescence and Primer Express software version 3.0 (Applied Biosystems, Foster City, CA, USA). Sense and antisense primer sequences were ordered (Choma Biotechnologies, Seongnam, Republic of Korea) to amplify amino peptidase (AP) (FW: CTCTCTCCTCAAAACCACATGAA; RV: AGTTCAGGGCCTTCTCATATTC), sucrase isomaltase (SI) (FW: CCTCCAGAACACAATCCCTATAC; RV: GGAGAGGTGAGATGGGATTAGA), peptide transporter 1 (PepT-1) (FW: CCTGGTCGTCTGCATCATATT; RV: TTCTTCTCATCCCTCATCGAACTG), and sodium glucose transport protein (SGLT-1) (FW: CATCCAGTCCATCACCAGTTAC; RV: CAATCAGGAAGCCGAGAATCA). The relative expression levels of mRNA were normalized to the endogenous control (beta-actin; FW: TTCGTTGCCGGTCCACACCC; RV: GCTTTGCACATGCCGGAGCC). All the steps were performed under open conditions with RNase.

#### 2.5.10. Statistical Analyses

The results of the chemical characterization of the flours were analyzed using Student’s *t*-test. For the in vivo study, data were first subjected to the Kolmogorov–Smirnov test to assess normality. As all variables followed a normal distribution, one-way analysis of variance (ANOVA) was applied, followed by the Newman–Keuls post hoc test to determine differences among means. The experimental design was completely randomized, with eight replicates per group (n = 8). Data are expressed as means ± standard deviation (SD), and results were considered statistically significant at *p* ≤ 0.05.

Spearman’s rank correlation coefficient was used to evaluate associations among body weight gain, body adiposity, short-chain fatty acid (SCFA) concentrations, fecal moisture content, fecal pH, cecal histomorphometric parameters, biochemical markers, and intake of resistant starch (RS), soluble fiber (SF), insoluble fiber (IF), and total phenolics (TP), with a significance level of *p* ≤ 0.05. All statistical analyses were performed using GraphPad Prism^®^ software, version 8.0 (GraphPad Software Inc., San Diego, CA, USA).

## 3. Results

### 3.1. Chemical Characterization of the Green Banana Pulp and Peel Flours

The concentration of proteins, lipids, total dietary fiber, and ash was higher in the PeF than in the PF (Table 2). On the other hand, the highest concentrations of total starch and resistant starch were observed in the PF (69.54 and 44.11 g per 100 g, respectively). Concerning the dietary fiber, the insoluble type (34.68 g per 100 g) constituted most of the chemical composition of PeF, while the PF showed considerable amounts of insoluble and soluble fibers (6.24 and 2.39 g/100 g, respectively). In addition, the total phenolic content was higher in the PeF when compared to the PF (7.60 and 3.80 mg GAE/g, respectively) (Table 2).

### 3.2. In Vivo Study

No statistical differences (*p* > 0.05) in food intake were observed among the experimental groups (Table 3). However, animals from the groups M1, M2, and P had significantly lower (*p* < 0.05) body weight gain and food efficiency ratios (FER) compared to those fed the control diet (SD group). In addition, it was observed that the SD group had higher (*p* < 0.05) adiposity compared to the M1, M2, and P groups. Resistant starch and soluble fiber consumption were higher (*p* < 0.05) in the animals fed the M1 and P diets compared to the animals fed the M2 diet. On the other hand, the insoluble fiber consumption was higher (*p* < 0.05) in the SD group compared to the M1, M2, and P groups.

**Figure 1:**
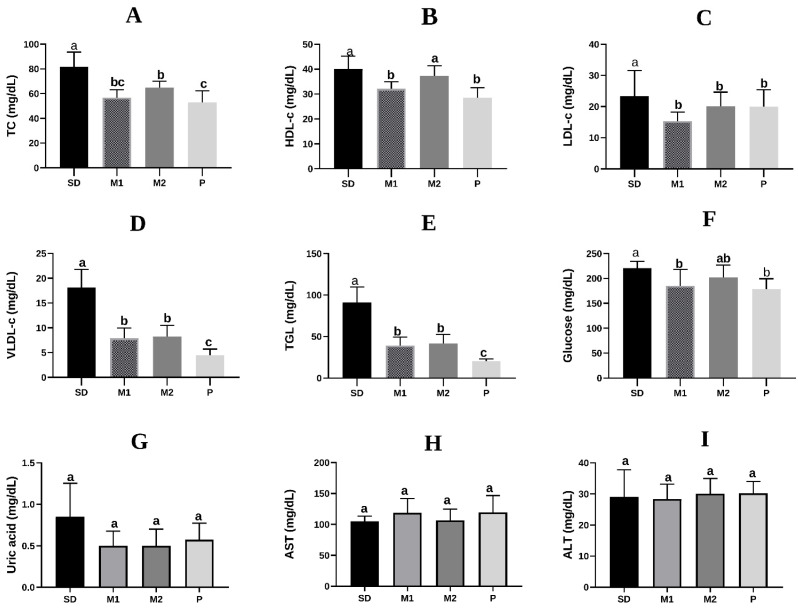
Effects of the consumption of green banana pulp flour and mixed green banana pulp and peel flours for 28 days on lipid profile (n = 8). (**A**) TC: total cholesterol; (**B**) HDL-c: high-density lipoprotein; (**C**) LDL-c: low-density lipoprotein; (**D**) VLDL-c: very low-density lipoprotein; (**E**) TGL: triglycerides; (**F**) glucose; (**G**) uric acid; (**H**) AST: alanine aminotransferase; (**I**) ALT: aspartate aminotransferase. SD: standard diet (AIN-93G); M1: standard diet + 90% of the PF and 10% of the PeF; M2: standard diet + 80% of the PF and 20% of the PeF; P: standard diet + 100% of the PF. Data were analyzed by one-way ANOVA. Means followed by different letters differ significantly, according to Newman–Keuls post hoc test (α = 0.05).

In terms of lipid profile, it was observed that the animals fed the M1, M2, and P groups had lower (*p* < 0.05) contents of total cholesterol, LDL-c, VLDL-c, and triglycerides compared to the control diet (Figure 1A,C–E). Furthermore, the M1 and P groups had a reduction (*p* < 0.05) in glycemic levels, while the M2 group did not significantly differ from the other groups (Figure 1F). AST and ALT enzyme activities, as well as uric acid levels (Figure 1G–I), did not differ (*p* > 0.05) among the experimental groups.

**Figure 2:**
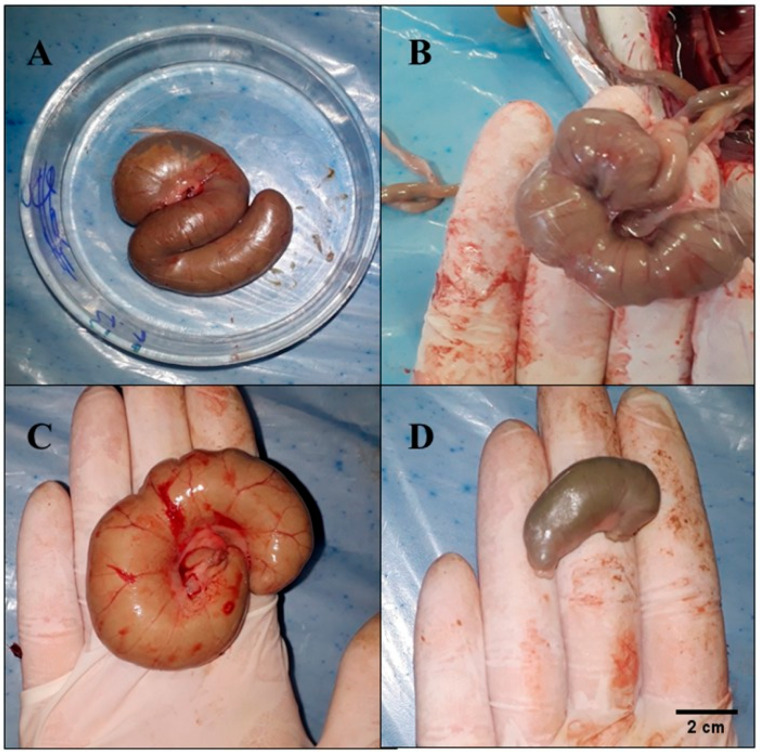
Effect of green banana pulp flour and mixed green banana pulp and peel flour intake on the cecum of young *Wistar* rats after 28 days of treatment (n = 8). (**A**) Cecum of animal from the M1 group (standard diet + 90% of the PF and 10% of the PeF); (**B**) cecum of animal from the M2 group (standard diet + 80% of the PF and 20% of the PeF); (**C**) cecum of animal from the P group (standard diet + 100% of the PF); and (**D**) cecum of animal from the SD group (standard diet only).

The cecum weight and cecum index were higher (*p* < 0.05) in the M1, M2, and P groups compared to the SD group (Table 4 and Figure 2). Animals fed the PF and PeF (M1, M2, and P groups) had higher (*p* < 0.05) fecal moisture content and lower fecal pH compared to animals fed the control diet (SD group). Furthermore, it was observed that M1, M2, and P showed higher (*p* < 0.05) concentrations of acetic acid compared to the SD group. There were no differences (*p* > 0.05) in the concentration of propionic and butyric acid, in the diameter of the muscle layers (circular and longitudinal), or in the number of goblet cells per crypt among the experimental groups. Crypt depth increased in all test groups, but crypt thickness increased only in the P group (Table 4).

**Figure 3:**
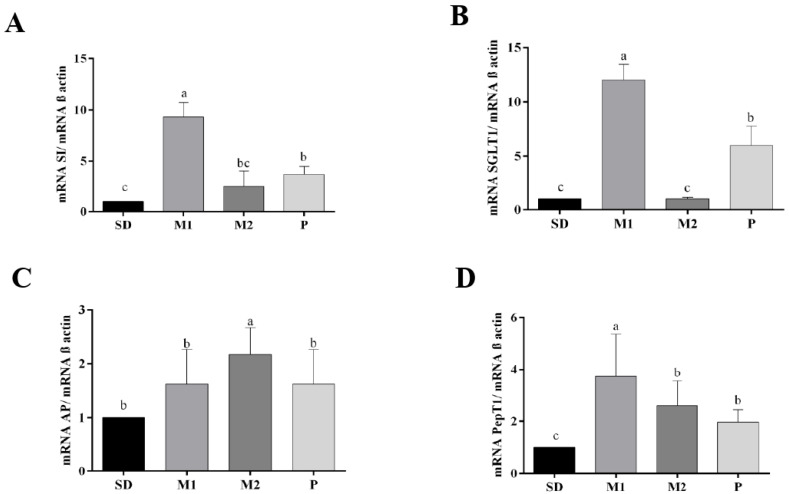
Effects of consumption of green banana pulp flour and mixed green banana pulp and peel flours for 28 days (n = 8) on the gene expression of brush border membrane functional proteins in the intestinal tissue, related to intestinal health. RT-qPCR analysis. (**A**) SI expression, (**B**) SGLT-1 expression, (**C**) AP expression, and (**D**) PepT-1 expression. SD: standard diet; M1: standard diet + 90% of the PF and 10% of the PeF; M2: standard diet + 80% of the PF and 20% of the PeF; P: standard diet + 100% of the PF; SI: sucrose isomaltase; SGLT1: sodium–glucose transport protein 1; AP: aminopeptidase; PepT1: peptide transporter. Data were analyzed by one-way ANOVA. Means followed by different letters differ significantly, according to the Newman–Keuls post hoc test (α = 0.05).

Regarding intestinal functionality, the M1 group had the highest PepT-1 gene expression, whereas the control group (SD) had the lowest (Figure 3D). In addition, M1 and P diet consumption significantly increased (*p* < 0.05) the SI and SGLT-1 gene expression compared to the control (SD) and M2 groups (Figure 3A,B). On the other hand, AP gene expression was significantly higher (*p* < 0.05) in the M2 group than in the other groups (M1, P, and SD) (Figure 3C).

#### Correlation Analysis

**Figure 4:**
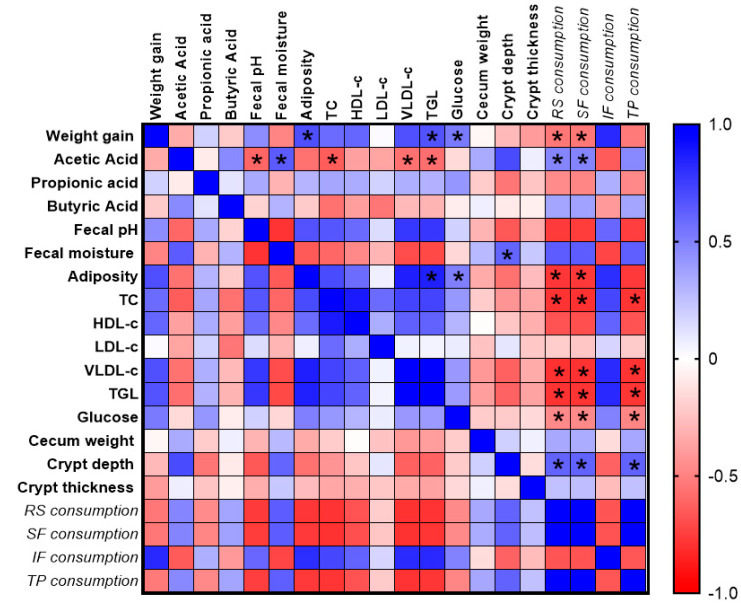
Heatmap of Spearman’s correlation analysis among dietary components, lipid profile, intestinal parameters, and histomorphometric measurements in *Wistar* rats. Blue and red colors represent positive and negative correlations, respectively, with color intensity proportional to the correlation coefficient (r), as indicated in the color scale. RS: resistant starch; SF: soluble fiber; IF: insoluble fiber; TP: total phenolics; TC: total cholesterol; LDL-c: low-density lipoprotein cholesterol; HDL-c: high-density lipoprotein cholesterol; VLDL-c: very low-density lipoprotein; TGL: triglycerides. * Indicates statistically significant difference (*p* < 0.05). Data represent n = 8/group (SD, M1, M2, and P groups).

Spearman’s correlation analysis was used to assess the relationships between body weight gain, adiposity, concentrations of SCFA, fecal moisture content, fecal pH, cecum histomorphometry analysis, biochemical parameters, resistant starch (RS) consumption, soluble fiber (SF) and insoluble fiber (IF) consumption, and total phenolic (TP) consumption (Figure 4). Negative correlations were observed between weight gain and RS and SF consumption (r = −0.538, *p* = 0.001 and r = −0.538, *p* = 0.002, respectively). The body adiposity was negatively correlated with the RS and SF consumption (r = −0.780, *p* < 0.001 and r = −0.767, *p* < 0.001, respectively) and positively correlated with the triglycerides (r = 0.852, *p* = 0.001) and body weight gain (r = 0.685, *p* < 0.001). Acetic acid concentration was positively correlated with the RS and SF consumption (r = 0.479, *p* = 0.006 and r = 0.483, *p* = 0.005, respectively) and fecal content moisture (r = 0.642, *p* < 0.001) and negatively correlated with the fecal content pH (r = −0.598, *p* = 0.001). Crypt depth was positively correlated with the fecal content moisture (r = 0.606, *p* = 0.004), RS, SF, and TP consumption (r = 0.610, *p* = 0.002; r = 0.615, *p* = 0.001; and r = 0.614, *p* = 0.001, respectively). Furthermore, a negative correlation was observed between glucose level and the RS, SF, and TP consumption (r = −0.469, *p* = 0.007; r = −0.468, *p* = 0.007; and r = −0.479, *p* = 0.006, respectively), and it was positive correlated with body weight gain (r = 0.524, *p* = 0.002) and adiposity (r = 0.503, *p* = 0.003). Total cholesterol, low-density lipoprotein cholesterol (LDL-c), very low-density lipoprotein (VLDL-c), and triglyceride (TGL) contents were negatively correlated with the consumption of RS (r = −0.789, *p* < 0.001; r = −0.799, *p* < 0.001; and r = −0.790, *p* < 0.001, respectively) and SF (r = −0.800, *p* < 0.001; r = −0.795, *p* < 0.001; and r = −0.786, *p* < 0.001, respectively) and TP (r = −0.786, *p* < 0.001; r = −0.791, *p* < 0.001; and r = −0.781, *p* < 0.001, respectively) and acetic acid concentration (r = −0.640, *p* = 0.001; r = −0.562, *p* < 0.001; and r = −0.572, *p* < 0.001, respectively) (Figure 4).

## 4. Discussion

Green banana pulp and peel flour showed high contents of resistant starch, specifically in the pulp flour. In addition, high contents of total dietary fiber and phenolic compounds were detected, specifically in peel flour. These observations suggest, as demonstrated in the current study, a positive impact of mixed green banana pulp and peel flours on body weight, adiposity, lipid profile, and intestinal morphology in *Wistar* rats. Thus, the present study indicated that the consumption of both green banana pulp flour and mixed green banana pulp and peel flours, associated with standard diet for 28 days, reduced body weight gain and adiposity. In addition, they increased the production of acetic acid and cecum weight, reduced the fecal pH, and increased fecal moisture, crypt depth, and the expression of intestinal functionality biomarkers. Further, green banana pulp flour (test group P) and mixed green banana pulp and peel flour (test groups M1 and M2) intake improved glucose homeostasis and lipid profile.

This study demonstrated that there were no significant differences (*p* > 0.05) in food intake among experimental groups, although a reduction in weight gain and adiposity in animals that consumed the green banana pulp flour and mixed green banana pulp and peel flours was observed (groups P, M1, and M2). These effects could be attributed to the higher consumption of dietary resistant starch and soluble fiber that are present in the flours, which correlated negatively with triglycerides, weight gain, and glucose levels (Figure 4). Resistant starch can improve lipid metabolism disorder and inhibit the accumulation of triglycerides in adipose tissue, since, after the intake of resistant starch, the fat synthesis rate is significantly reduced, which involves body weight control [23].

P, M1, and M2 groups demonstrated higher fecal moisture, acetic acid concentration, and cecum weight and reduced fecal pH. The consumption of resistant starch and dietary fibers was positively correlated with acetic acid production, bacterial populations, and fecal moisture, and it was negatively correlated with fecal content pH. Resistant starch is not digested in the small intestine and is be used as a substrate for the gut microbiota fermentation activity. The increased bacterial fermentation had multiple health benefits to the host, including production of SCFA and consequently reducing intestinal pH, which may increase mineral solubility and absorption [24,25,26].

In agreement with these observations, animals in the groups P, M1, and M2 demonstrated increased crypt depth, which was positively correlated with fecal moisture, and higher resistant starch, soluble fiber, and total phenolic dietary consumption. The observed morphological effects can be attributed to the potential contribution of dietary resistant starch and dietary fibers in general to the increased proliferation of intestinal enterocytes that resulted in hyperplasia and/or hypertrophy of the brush border membrane (BBM) [25]. This cellular proliferation can also be related to the higher content of phenolic compounds in flours, which can lead to alteration in intestinal morphology [27,28]. Mishima et al. (2023) [24] observed that the chia phenolic extract increased the gene expression of SI and crypt depth.

Furthermore, the expression of BBM functional gene PepT-1 was higher in the groups P, M1, and M2 compared to the control group. The M1 and P diets increased the expression of SGLT-1 and SI relative to the M2 and control group and decreased the expression of the AP gene compared to the M2 diet. These findings further indicate that dietary resistant starch, fiber, and phenolic compounds lead to the increased production of secondary metabolites as SCFA, specifically acetic acid, which acts as a substrate to the gut microbiota, promoting improvement in BBM. Moreover, acetic acid improves BBM by enhancing nutrient transport, stimulating digestive enzyme activity, and protecting structural integrity [29]. PepT-1 is a protein that has the function of moving peptides from the lumen of the small intestine to the enterocyte, and SGLT-1 is responsible for dietary glucose absorption, and it is highly expressed on the BBM of the villi of enterocytes [30]. In addition, the SI gene is a disaccharidase located on the duodenal BBM that hydrolyzes carbohydrates for absorption, and AP gene is an enzyme that cleaves amino acids from the N-terminus of peptides [31,32]. Thus, green banana flours showed a positive impact on BBM enzymes and nutrient absorption.

In our study, a decrease in blood glucose levels was observed in the groups fed the green banana pulp flour and a mixed green banana pulp and peel flour (M1 and P diet), which was negatively correlated with the dietary consumption of resistant starch, fiber, and total phenolics. Dietary fiber and resistant starch can reduce the rate of digestion and enzymatic activity of amylase, thus preventing the increase of blood glucose levels and insulin and the decrease of post-meal plasma glucose [33]. Furthermore, phenolic compounds can partially inhibit the activity of α-amylase and α-glucosidase during enzymatic carbohydrate hydrolysis, reducing the absorption rate and consequently improving the glycemic response [34], as observed in animals fed sorghum, a cereal rich in antioxidants and dietary fiber [23].

Additionally, our results demonstrated improvement in the lipid profile of animals fed the green banana pulp flour and mixed green banana pulp and peel flours, decreasing total cholesterol, LDL-c, VLDL-c, and triglycerides contents in blood. These changes can be explained by a negative correlation among resistant starch, dietary fiber, and phenolic compound consumption and acetic acid production, by the gut microbiota, with lipid profile. Whole foods, which are sources of resistant starch and other dietary fibers, exert hypocholesterolemic effects because they act on the hepatic metabolism [35] and, therefore, increase the excretion of bile acid fecal, decreasing the entero-hepatic reabsorption. Thus, more endogenous cholesterol is used to synthetize biliary acid, reducing endogen cholesterol, as the resistant starch and dietary fiber can reduce the absorption of cholesterol and circulating lipids. In addition, the increase in acetic acid concentration might be associated with the decreased lipid profile parameters in the blood. The acetic acid produced by bacterial fermentation enters the bloodstream and reaches the liver via the portal vein, where it is converted to acetyl coenzyme A and can be used as an energy source or as substrate for the synthesis of long-chain fatty acids and cholesterol [36]. Our study had many strengths, such as a robust experimental design; adequate sample size, novelty, and relevance; and relevant outcomes. A limitation would be a lack of some analyses, such as fecal DNA sequencing to evaluate how the consumption of the green banana flours modulated the gut microbiota. However, we have been working on a follow-up study where more robust information on intestinal health can be obtained.

## 5. Conclusions

The consumption of mixed green banana pulp and peel flours, specifically the mixture M1 (90% of pulp flour and 10% of peel flour), a source of resistant starch, other dietary fibers, and phenolic compounds, demonstrated potential to reduce body weight gain and adiposity, glucose levels, and blood lipid profile in *Wistar* rats. Furthermore, the flours’ intake contributed to increased acetic acid concentration and fecal moisture, decreased cecum content pH, and improved intestinal gene expression and crypt structure. Thus, the mixed green banana pulp and peel flour is a promising basic food product ingredient, with demonstrated health benefits.

## Figures and Tables

**Table 1:** Nutritional composition of the experimental diets (g per 100 g).

Ingredients	Experimental Diets			
	SD	M1	M2	P
Casein *	16.16	14.27	14.56	13.77
Mixed PF + PeF (90:10)	-	42.65	-	-
Mixed PF + PeF (80:20)	-	-	33.74	-
Green banana pulp flour	-	-	-	57.94
Dextrinized starch	13.20	13.20	13.20	6.59
Sucrose	10.00	10.00	10.00	10.00
Soybean oil	7.00	6.44	6.33	6.65
Microcrystalline cellulose	5.00	-	-	-
Mineral mix	3.50	3.50	3.50	3.50
Vitamin mix	1.00	1.00	1.00	1.00
_L_-Cystine	0.30	0.30	0.30	0.30
Choline bitartrate	0.25	0.25	0.25	0.25
Corn starch	43.59	8.39	17.12	-
**Composition of diets (%)**				
Protein	16.40	16.70	16.60	16.70
Carbohydrate	67.70	66.70	66.90	66.50
Lipid	16.00	16.60	16.50	16.80
Caloric density (Kcal/g)	3.95	3.79	3.81	3.75

SD: standard diet; M1: 90% of the PF and 10% of the PeF; M2: 80% of the PF and 20% of the PeF; P: 100% of the PF. * Considering that casein had 81% purity.

**Table 2:** Chemical composition of the pulp and peel flours of green banana (*Musa* spp.).

Components	Flours	
	PF	PeF
Moisture (g/100 g)	6.15 ± 0.15 ^b^	7.40 ± 0.13 ^a^
Protein (g/100 g)	3.33 ± 0.48 ^b^	5.80 ± 0.00 ^a^
Lipids (g/100 g)	0.61 ± 0.05 ^b^	7.53 ± 0.02 ^a^
Ash (g/100 g)	2.23 ± 0.04 ^b^	8.41 ± 0.11 ^a^
Total starch (g/100 g)	69.54 ± 0.75 ^a^	24.04 ± 0.00 ^b^
Resistant starch (g/100 g)	44.11 ± 0.66 ^a^	17.89 ± 0.40 ^b^
**Total dietary fiber (g/100 g)**	8.63 ± 0.62 ^b^	39.57 ± 1.23 ^a^
Soluble fiber (g/100 g)	2.39 ± 0.65 ^b^	4.89 ± 0.64 ^a^
Insoluble fiber (g/100 g)	6.24 ± 0.03 ^b^	34.68 ± 0.59 ^a^
Total phenolics (mg GAE per g)	3.80 ± 0.002 ^b^	7.60 ± 0.004 ^a^

GAE: gallic acid equivalent; PF: green banana pulp flour; PeF: green banana peel flour. Results were expressed as mean ± standard deviation. Means followed by the same letter in the rows (PF versus PeF) do not differ by the Student’s *t*-test (α = 0.05).

**Table 3:** Weight gain, adiposity, food intake, FER, and consumption of resistant starch and soluble and insoluble fibers by the animals.

Parameters	Groups			
	SD	M1	M2	P
Weight gain (g)	168.57 ± 9.59 ^a^	153.84 ± 10.24 ^b^	154.02 ± 7.62 ^b^	146.95 ± 6.37 ^b^
Adiposity (%)	4.27 ± 0.77 ^a^	2.45 ± 0.41 ^b^	2.90 ± 0.39 ^b^	1.39 ± 0.21 ^c^
Food intake (g/week)	118.93 ± 7.50 ^a^	122.18 ± 6.35 ^a^	120.19 ± 8.04 ^a^	120.67 ± 7.70 ^a^
FER	35.47 ± 1.38 ^a^	31.48 ± 1.44 ^bc^	32.62 ± 1.31 ^b^	30.32 ±1.77 ^c^
**Total consumption (g/day)**				
Resistant starch (g/day)	-	3.09 ± 0.16 ^b^	2.25 ± 0.15 ^c^	4.41 ± 0.28 ^a^
Soluble fiber (g/day)	-	0.20 ± 0.01 ^b^	0.17 ± 0.01 ^c^	0.24 ± 0.02 ^a^
Insoluble fiber (g/day)	0.85 ± 0.05 ^a^	0.68 ± 0.04 ^b^	0.71 ± 0.05 ^b^	0.62 ± 0.04 ^c^

Results are expressed as mean ± standard deviation, n = 8/group. SD: standard diet; M1: 90% of the PF and 10% of the PeF; M2: 80% of the PF and 20% of the PeF; P: 100% of the PF. The data were analyzed by one-way ANOVA. Means followed by different letters in the same row differ significantly, according to Newman–Keuls post hoc test (α = 0.05).

**Table 4:** Cecum weight, cecum index, fecal pH, fecal moisture, short-chain fatty acids, and histomorphometry characteristics of the cecum of animals.

Parameters	Groups			
	SD	M1	M2	P
Cecum weight (g)	3.90 ± 0.39 ^c^	15.09 ± 2.10 ^a^	10.27 ± 2.97 ^b^	17.19 ± 3.85 ^a^
Cecum index (%)	2.33 ± 0.29 ^c^	10.16 ± 1.11 ^a^	6.55 ± 1.67 ^b^	13.30 ± 2.64 ^a^
Fecal moisture (%)	34.80 ± 7.31 ^c^	52.98 ± 4.95 ^b^	57.11 ± 4.33 ^b^	65.37 ± 7.12 ^a^
Fecal pH	8.32 ± 0.38 ^a^	6.80 ± 0.68 ^b^	6.29 ± 0.78 ^b^	5.17 ± 0.34 ^c^
**Short**-**chain Fatty Acids (mM)**				
Acetic acid	4.86 ± 0.93 ^b^	8.93 ± 3.196 ^a^	8.14 ± 0.96 ^a^	8.19 ± 1.71 ^a^
Butyric acid	1.33 ± 1.33 ^a^	2.17 ± 0.69 ^a^	2.12 ± 1.69 ^a^	2.02 ± 0.96 ^a^
Propionic acid	1.90 ± 0.34 ^a^	1.87 ± 0.09 ^a^	1.80 ± 0.44 ^a^	1.37 ± 0.59 ^a^
Crypt depth (μm)	130.50 ± 15.30 ^b^	166.10 ± 22.13 ^a^	176.50 ± 14.77 ^a^	192.80 ± 29.54 ^a^
Crypt thickness (μm)	37.98 ± 2.12 ^b^	38.50 ± 3.70 ^b^	38.59 ± 2.07 ^b^	46.52 ± 8.99 ^a^
Number of goblet cells	19.82 ± 2.70 ^a^	20.11 ± 2.56 ^a^	21.82 ± 2.98 ^a^	21.28 ± 3.00 ^a^
CML (μm)	58.67 ± 11.86 ^a^	73.29 ± 6.62 ^a^	69.66 ± 13.57 ^a^	69.44 ± 5.51 ^a^
LML (μm)	28.50 ± 4.52 ^a^	30.88 ± 3.03 ^a^	30.27 ± 2.71 ^a^	29.41 ± 6.81 ^a^

Results were expressed as mean ± standard deviation, n = 8/group. SD: standard diet; M1: 90% of the PF and 10% of the PeF; M2: 80% of the PF and 20% of the PeF; P: 100% of the PF; CML: circular muscle layer; LML: longitudinal muscle layer. The data were analyzed by one-way ANOVA. Means followed by different letters in the same row differ significantly, according to the Newman–Keuls post hoc test (α = 0.05).

## Data Availability

Data described in the manuscript, code book, and analytic code will be made available upon request.
